# Cost of illness in a super-aged society—comparison of breast, lung, and prostate cancer in Japan

**DOI:** 10.1186/s12877-022-03683-3

**Published:** 2022-12-14

**Authors:** Kunichika Matsumoto, Yosuke Hatakeyama, Kanako Seto, Ryo Onishi, Koki Hirata, Yinghui Wu, Tomonori Hasegawa

**Affiliations:** 1 Department of Social Medicine, Toho University School of Medicine, 5-21-16 Omori-Nishi, Ota-Ku, Tokyo, 143-8540 Japan; 2 School of Nursing, Shanghai Jiao Tong University, 227 South Chongqing Road, Shanghai, People’s Republic of China

**Keywords:** Cost of illness, Aging, Cancer, Health economics, Japan

## Abstract

**Background:**

Aging increases the disease burden because of an increase in disease prevalence and mortality among older individuals. This could influence the perception of the social burden of different diseases and treatment prioritization within national healthcare services. Cancer is a disease with a high disease burden in Japan; however, the age-specific frequency and age-specific mortality rates differ according to site. In this study, we evaluated the relationship between the aging of the Japanese society and the disease burden by comparing the features of three cancers with different age-specific frequency rates in Japan. Furthermore, we made projections for the future to determine how the social burden of these cancers will change.

**Methods:**

We calculated the social burden of breast, lung, and prostate cancers by adding the direct, morbidity, and mortality costs. Estimates were made using the cost of illness (COI) method. For future projections, approximate curves were fitted for mortality rate, number of hospital admissions per population, number of outpatient visits per population, and average length of hospital stay according to sex and age.

**Results:**

The COI of breast, lung, and prostate cancers in 2017 was 903.7, 1,547.6, and 390.8 billion yen, respectively. Although the COI of breast and prostate cancers was projected to increase, that of lung cancer COI was expected to decrease. In 2017, the average age at death was 68.8, 76.8, and 80.7 years for breast, lung, and prostate cancers, respectively.

**Conclusions:**

Patients with breast cancer die earlier than those with other types of cancer. The COI of breast cancer (“young cancer”) was projected to increase slightly because of an increase in mortality costs, whereas that of prostate cancer (“aged cancer”) was projected to increase because of an increase in direct costs. The COI of lung cancer (“aging cancer”) was expected to decrease in 2020, despite the increase in deaths, as the impact of the decrease in human capital value outweighed that of the increase in deaths. Our findings will help prioritize future policymaking, such as cancer control research grants.

## Background

Japan’s population is aging rapidly. According to the United Nations, the proportion of individuals aged ≥ 65 years, has surpassed 28% and may reach 30.9% by 2030 [1]. In an aging population, the increasing prevalence and mortality of diseases common among older individuals exert a great influence on the disease burden. This, in turn, can affect the perceived social burden of these diseases, leading to prioritization changes in the policies of national healthcare services. Cancer, which is the leading cause of death in Japan since 1981, even if treatable, is linked to a decline in the current and future performance of activities of daily living (ADLs), and it imposes a significant social burden [2]. Cancer is also a disease for which prevention is important, in addition to other chronic diseases that cause other ADL-related challenges [3, 4].

In Japan, national medical expenditures have been increasing significantly—from 30.1 trillion yen (5.7% of GDP) in 2000 to 44.4 trillion yen (7.9% of GDP) in 2019. In Japan’s medical system, almost all government-approved therapeutic and diagnostic methods are covered by public medical insurance; thus, there are no prioritized restrictions on these methods [5]. However, the government also devotes significant resources to disease control, such as providing many subsidies to promote medical research. For these policies, the prioritization of evidence-based policymaking is needed. It makes sense to allocate research and countermeasure funds to diseases with a high social burden, as this would improve population welfare while also satisfying efficient budget allocation. In this study, we compared the social burden of cancer according to site from the perspective of age-specific incidence. In Japan, where the birthrate has been rapidly declining and the population has been aging, the demographic structure has also been changing dramatically, and we believe that cancers with different incidence rates by age group differ not only in changes in social burden but also in the composition of the social burden.

Particularly, we assessed the relationship between aging and disease burden by comparing three main cancer types that occur in Japan: breast, prostate, and lung cancers. The crude mortality rate (total number of deaths per year per 1,000 people) continues to increase [6]. The trend in age-specific mortality as well as age-specific incidence rates depend strongly on the type of cancer. We analyzed the trends in social burden, and the factors influencing it, based on the frequency of cancer with age; whether it is more common in younger patients, increases in frequency with age, or is more common in elderly patients. Thus, breast cancer (International Statistical Classification of Diseases and Related Health Problems 10th Revision (ICD10): C50) was identified as a *young cancer* because of the low average age of incidence and death. Lung cancer (ICD10: C33–C34) was identified as an *aging cancer* because its incidence and mortality rise dramatically with age. Prostate cancer (ICD10: C61) was identified as an *aged cancer* because the average age at death from this disease is similar to the average Japanese life expectancy.

Although the incidence of breast cancer been increasing in Western countries recently, its age-adjusted incidence rate has been declining [7–9]. However, in Japan, both the incidence rate and the age-adjusted incidence rate have been increasing [10, 11]. Moreover, breast cancer occurs at an earlier age than other forms of cancer, with a peak incidence occurring in the late 40 s and 60 s. However, in the United States and Europe, the incidence of breast cancer peaks after 60 years of age [12, 13]. In Japan, lung cancer is the leading cause of death in men and the second-leading cause of death in women. Although the age-adjusted mortality of lung cancer has decreased recently, the crude mortality and number of deaths have increased because of its high incidence rate among the elderly [11, 13–16]. In Japan, the number of patients diagnosed with prostate cancer or dying from it has increased recently; the proportion of patients with prostate cancer among all patient with any type of cancer increased from 3.3% in 1996 to 5.8% in 2020 [10, 11]. Prostate cancer is typically diagnosed in elderly patients. More than 90% of patients with prostate cancer are aged > 65 years.

Few studies have attempted to estimate the economic burden of cancer in Japan [17, 18]. Most assessed the direct medical costs only at single timepoints (There are few time-series analysis). However, such an approach is insufficient to compare the social burden among different cancer types in term of age of incidence and death. No previous studies have investigated the chronological trends in the social burden of cancers, including indirect cost (IC).

The cost-of-illness (COI) method was first used in the analysis of mental diseases by Malzeberg [19], and it was further developed by Rice [20, 21] at the National Center for Health Statistics in the United States. Rice conducted successive case studies and promoted formalizing the method. Basically, the COI includes both direct costs (DC) and IC, with IC including morbidity costs (MbC) and mortality costs (MtC). DC is the expense of a specific disease, MbC is the loss of labor value during hospitalization or a hospital visit, and MtC is the loss of human capital caused by early death. The COI method has been used for macroscopic analysis rather than microscopic analysis. In macroscopic analysis, the COI can be calculated as a “top–down” method using aggregated statistics of a nation or society. In countries with available national data, calculating the COI of major diseases is possible. The effect of policies can be evaluated for disease control in monetary terms, and this evaluation contributes to prioritizing policies.

The COI has been widely used because of such characteristics. The COI method has influenced the decision making of the government in the United States for more than 30 years. For example, Kirschstein et al. reported the results of COI calculation of major diseases to the United States Congress [22]. The studies on the COI of tobacco-caused diseases in Medicaid by Miller et al. and Wamer et al. were used to revise state laws about the tobacco industry [23, 24]. The COI study on trauma by Max et al. motivated the Centers for Disease Control and Prevention to launch a trauma center [25]. Outside the United States, the COI method was used by the National Institute for Clinical Excellence in the United Kingdom and the Pharmaceutical Benefits Schema in Australia, among other cited examples [26]. Tarricone reported that COI studies were among the most common economic studies in healthcare in Italy and abroad, and they were commonly used by organizations such as the World Bank, the World Health Organization, and the United States National Institutes of Health [20].

This method for measuring the social burden of illness has evolved but has also generated a lot of criticism [27, 28]. Particularly, a group led by Michael Drummond of the University of York has criticized COI research for its economic evaluation of healthcare [29–31]. Drummond defines the economic assessment of healthcare as “a comparative analysis of healthcare programs in terms of both cost and effectiveness.” He believes that two perspectives are needed. First, healthcare programs should be evaluated and compared with other alternatives. Second, the costs and outcomes of the alternatives should be considered. COI research does not compare healthcare alternatives; instead, it analyzes costs only. Therefore, Drummond concluded that COI studies are not complete economic assessments.

In contrast, COI research obtains methodological superiority from the recognition that it is different from other economic evaluations [30, 32–34]. Particularly, the COI method has the advantage of being relatively simple to calculate using easily obtainable data. The COI can also be estimated according to demographic group. Thus, it represents an appropriate method for studying the relationship between social aging and disease burden. We have also previously COI to estimate the social burden of different diseases [35–43].

With this background, we conducted COI research using a top-down, *human capital* approach. Estimates of social burden are more accurate if they are based on macrodata (e.g., census data). Some previous COI studies have adopted a *friction cost* approach, rather than a human capital approach. However, the friction cost approach neglects social perceptions and only reflects employers’ views [44]. The human capital approach has been criticized for overestimating the COIs; however, this is irrelevant for comparative purposes. We estimated the COIs of three major cancers since 1996 and the projected future COIs up to 2029. We estimated and modeled the economic burden associated with breast, lung, and prostate cancers, and analyzed the effects of population aging by comparing the COIs among these cancers. Understanding trends in the social burden of the three major cancers will be useful in prioritizing future policy development, such as subsidies for cancer control research.

## Methods

We used governmental statistics to estimate the COI using a “top-down” method (Table 1). The government statistics on population and mortality are complete census, whereas the others are sample surveys of the entire population. We estimated the disease burden from a societal perspective and estimated the COI generated per year every 3 years in a time series for future estimates. We used the same estimation method that has been developed by Rice et al. and that we had previously used to estimate the COIs of other diseases [35–43].

**Table 1:** Sources of data on the cost of illness for the three cancer types

Source	Issuer	Purpose
Statistics of Medical Care Activities in Public Health Insurance	Ministry of Health, Labour and Welfare	To determine the direct cost of each cancer
Basic Survey on Wage Structure	Ministry of Health, Labour and Welfare	To calculate labor value
Labour Force Survey	Ministry of Internal Affairs and Communications	To calculate labor value
Estimates of Monetary Valuation of Unpaid Work	Cabinet Office	To calculate labor value
Vital Statistics	Ministry of Health, Labour and Welfare	To evaluate the number of deaths
Patient Survey	Ministry of Health, Labour and Welfare	To distinguish the number of patients, total person-days of outpatient visits, and average length of hospital stay
Population Projections for Japan	National Institute of Population and Social Security Research in Japan	To refer future population

The COI is estimated as the sum of DC and IC, with IC being divided into MbC and MtC [35], using the following formula:

$$\mathrm{COI }=\mathrm{DC }+\mathrm{MbC }+\mathrm{MtC}.$$

DCs are the medical costs directly related to the disease, including costs associated with treatment, hospitalization, testing, and drugs. We determined the annual medical costs from the total medical expenses reported by the Survey of National Medical Care Insurance Services. These data relate to major forms of cancer but do not include prostate cancer. For prostate cancer, DC (hospitalization and outpatient costs) was estimated as follows: the hospitalization costs for each year were calculated from the hospitalization costs for prostate cancer in 2005, as reported by the Central Social Insurance Medical Council, adjusted for the general rate of increase in hospitalization expenses. Outpatient costs were calculated by multiplying the outpatient cost per day (for all cancers) by the total number of person-days of outpatient visits. IC consists of the opportunity costs resulting from disease or death. We calculated the MbC and MtC using the following equations:

$$\mathrm{MbC }=\mathrm{ TOVy }\times \mathrm{ LVd}/2 +\mathrm{ THD }\times \mathrm{ LVd}$$

$$\mathrm{MtC }=\mathrm{ NDy }\times \mathrm{ LVl}$$

where TOVy is the total person-days dedicated to outpatient visits, LVd is the one day labor value per person, THD is the total person-days of hospitalization, NDy is the number of deaths, and LVl is the lifetime labor value per person.

We calculated the TOVy and THD in 5-year age groups, based on the Japanese government’s 3-yearly Patient Survey. We determined labor values, in 5-year age groups, based on data from the Basic Survey on Wage Structure, Labor Force Survey, Estimates of Monetary Valuation of Unpaid Work, and Evaluations of Domestic Labor. We calculated the LVl by summing the potential future income had the patient survived. LVl was calculated at its current value, with a standard discount rate of 2% [45]. We calculated the MbC by assuming a loss of 1 day of labor value per day of hospitalization and of a half-day per outpatient visit. We calculated the number of deaths per cancer type for each 5-year age group, based on the Ministry of Health, Labor, and Welfare’s Vital Statistics.

We calculated the LVd and THD as follows:

$$\mathrm{LVd }= \left(\mathrm{Iy }+\mathrm{ ULVy}\right) / 365$$

$$\mathrm{THD }=\mathrm{ HPy }\times \mathrm{ ALOS}$$

where Iy is the annual income per person, ULVy is the annual monetary valuation of unpaid work per person, HPy is the annual number of hospitalized patients, and ALOS is the average length of hospital stay. Because the three cancer types have different age-specific incidence rates, not only the amount of COI per capita but also the percentages of the DC, MbC, and MtC that constitute the COI are expected to differ significantly.

For future predictions, we used the methods applied to other diseases in previous studies [35–41]. The potential effects of changes in population and age structure were considered. We estimated whether the rates of variation in health-related indicators have changed over the past 21 years (i.e., mortality rate, number of outpatient visits per population, number of hospitalizations per population, and average length of hospital stay). First, we obtained a trend line (logarithmic/exponential approximation and linear regression) for each indicator since 1996. We selected the regression line with the highest coefficient of determination for each parameter, and extrapolated the data to estimate the values for 2020, 2023, 2026, and 2029 according to sex and 5-year age groups. For future life expectancy and labor value, we used 2017 data. We also used data from population estimates published by the Ministry of Internal Affairs and Communications for 1996–2017. The data for 2020–2029 were taken from Population Statistics of Japan, published by the National Institute of Population and Social Security Research.

Linear regression often returned negative estimated values for some parameters. Therefore, we set a minimum value in our calculations. For the mortality rate, number of outpatient visits per population, and number of hospitalizations per population, we used the closest previous positive value whenever the line returned a negative value. For ALOS, we selected the lowest ALOS recorded as the minimum value (for each type of cancer) from any of the countries of the Organization for Economic Co-operation and Development.

Regarding the potential future labor value, we conducted a sensitivity analysis for the discount rate. Thus, the base case discount rate was 2%, and our analyses included a discount rate of 0%–5%.

We calculated the rates of contribution to overall COI variation of the DC, MbC, and MtC, as follows:

$$\frac{{Cost}_{t}^{i}-{Cost}_{0}^{i}}{{COI}_{t}-{COI}_{0}}$$

${Cost}_{t}^{i}$: DC, MbC, and MtC at year t

${Cost}_{0}^{i}$: DC, MbC, and MtC costs at the baseline year

$CO{I}_{t}$: COI at year t

$CO{I}_{0}$: COI at the baseline year.

This study was conducted in accordance with the Consolidated Health Economic Evaluation Reporting Standards 2022 [46].

## Results

### COI for 1996–2014

Table 2 shows the trends for the COI and health-related indicators for the period 1996–2017. The COIs of breast, lung, and prostate cancers in 1996 were 440.3, 969.6, and 139.6 billion yen, respectively. In 2017, they increased to 903.7, 1,547.6, and 390.8 billion yen, respectively. During this period, the annual percent change (APC) increased by 3.5% for breast cancer, 2.3% for lung cancer, and 5.0% for prostate cancer. The MtC represented the most important COI component for breast (65.6–76.0%) and lung (70.8–84.3%) cancers. For prostate cancer, the DC was the main COI component (49.2%–71.5%). The contribution ratios of the DC, MbC, and MtC to the total increase in the COI were + 39.2, + 5.1, and + 55.7%, respectively, for breast cancer; + 49.1, + 2.8, and + 48.2%, respectively, for lung cancer; and + 83.9, + 4.9, and + 11.1%, respectively, for prostate cancer. MtC was the main COI contributor for breast cancer, whereas the DC was the main contributor for lung and prostate cancers. Approximately 80% of the increase in the COI for prostate cancer was related to the DC.

**Table 2:** Cost of illness trends for the three cancer types

		1996	1999	2002	2005	2008	2011	2014	2017
Population (thousand persons)		125,864	126,686	127,435	127,768	127,692	127,799	127,082	126,706
% of individuals aged ≥ 65 years		15.1%	16.7%	18.5%	20.2%	22.1%	23.3%	26.0%	27.7%
Breast Cancer	Number of deaths (persons)	7962	8949	9675	10,808	11,889	12,791	13,322	14,423
	[% of individuals aged ≥ 65 years]	36.5%	38.0%	40.8%	44.0%	48.8%	52.1%	58.7%	63.6%
	Number of incidence (persons)	30,713	35,183	41,960	50,695	65,085	81,319	87,202	104,379
	[% of individuals aged ≥ 65 years]	27.9%	30.1%	32.0%	33.4%	36.0%	36.6%	42.0%	45.5%
	Crude mortality/incidence rate	25.9%	25.4%	23.1%	21.3%	18.3%	15.7%	15.3%	13.8%
	Average age at incidence (years)	56.3	57.4	58.2	59.0	59.5	60.0	60.9	61.9
	Average age at death (years)	60.4	61.3	62.3	63.6	65.0	66.3	67.6	68.8
	Direct cost (billion yen)	72.2	124.9	146.8	185.4	168.6	166.3	217.0	254.1
	Morbidity cost (billion yen)	33.3	40.1	49.0	43.7	47.2	48.4	49.3	56.9
	Mortality cost (billion yen)	334.7	362.2	438.0	459.1	480.0	533.8	523.8	592.7
	% of individuals aged ≥ 65 years	10.1%	10.5%	13.4%	14.6%	17.5%	20.9%	24.2%	31.0%
	Mortality cost per person (million yen)	42.0	40.5	45.3	42.5	40.4	41.7	39.3	41.1
	COI (billion yen)	440.3	527.2	633.7	688.3	695.7	748.5	790.1	903.7
Lung Cancer	Number of deaths (persons)	36,931	41,350	50,643	50,360	55,143	58,876	73,389	73,883
	[% of individuals aged ≥ 65 years]	76.9%	79.3%	81.4%	81.1%	82.5%	83.9%	86.8%	90.2%
	Number of incidence (persons)	59,741	65,967	73,635	83,881	97,275	111,858	114,550	124,510
	[% of individuals aged ≥ 65 years]	74.5%	75.6%	76.8%	75.8%	77.8%	77.5%	81.1%	83.9%
	Crude mortality/incidence rate	61.8%	62.7%	68.8%	60.0%	56.7%	52.6%	64.1%	59.3%
	Average age at incidence (years)	70.5	71.0	71.6	71.8	72.5	72.6	73.0	73.8
	Average age at death (years)	71.5	72.3	73.7	73.8	74.4	75.1	75.7	76.8
	Direct cost (billion yen)	116.3	186.1	208.6	262.7	244.3	278.2	317.3	399.9
	Morbidity cost (billion yen)	36.2	47.6	53.8	58.3	58.0	56.1	55.3	52.3
	Mortality cost (billion yen)	817.1	807.7	990.3	982.4	1050.6	1152.3	1131.8	1095.4
	% of individuals aged ≥ 65 years	31.1%	33.2%	41.2%	40.4%	43.3%	50.1%	53.8%	63.7%
	Mortality cost per person (million yen)	22.1	19.5	19.6	19.5	19.1	19.6	15.4	14.8
	COI (billion yen)	969.6	1041.5	1252.7	1303.4	1352.9	1486.6	1504.5	1547.6
Prostate Cancer	Number of deaths (persons)	6009	7005	8105	9264	9989	10,823	11,507	12,869
	[% of individuals aged ≥ 65 years]	91.4%	92.8%	93.0%	93.9%	94.9%	95.2%	96.2%	97.1%
	Number of incidence (persons)	14,077	17,056	29,345	42,997	51,534	78,728	74,459	91,215
	[% of individuals aged ≥ 65 years]	87.7%	88.0%	86.5%	84.3%	84.1%	82.8%	86.1%	88.1%
	Crude mortality/incidence rate	42.7%	41.1%	27.6%	21.5%	19.4%	13.7%	15.5%	14.1%
	Average age at incidence (years)	74.5	74.4	73.6	72.6	72.8	72.6	73.1	74.0
	Average age at death (years)	77.2	77.7	77.9	78.3	78.8	79.3	79.9	80.7
	Direct cost (billion yen)	68.7	83.9	97.0	164.2	195.9	219.3	253.1	279.6
	Morbidity cost (billion yen)	6.3	7.9	10.7	16.9	17.5	17.9	17.7	18.7
	Mortality cost (billion yen)	64.6	70.5	80.0	86.0	87.2	89.0	88.7	92.5
	% of individuals aged ≥ 65 years	66.8%	70.7%	70.2%	73.0%	74.8%	76.6%	79.8%	82.0%
	Mortality cost per person (million yen)	10.7	10.1	9.9	9.3	8.7	8.2	7.7	7.2
	COI (billion yen)	139.6	162.3	187.6	267.0	300.6	326.2	359.5	390.8

Source of population data: Ministry of Internal Affairs and Communications “Population Estimates.”

Source of the number of cancer deaths: “Vital Statistics.”

Source of the number of incidence cases: Center for Cancer Control and Information Services, National Cancer Center, Japan

Average age at incidence: Calculated according to the number of incidence

Average age at death: Calculated according to the number of deaths, sex, age (5-year-old age-grade), and cause of death in “Vital Statistics.”

Between 1996 and 2017, the number of deaths increased for each type of cancer: from 7,962 to 14,423 (APC, 2.9%) for breast cancer, from 36,931 to 73,883 (APC, 3.4%) for lung cancer, and from 6,009 to 12,869 (APC, 3.7%) for prostate cancer. The death rate for prostate cancer increased more dramatically than that for any other cancer type. The proportion of deceased individuals older than 65 years increased consistently for each cancer type: from 36.5 to 63.6% for breast cancer, from 76.9 to 90.2% for lung cancer, and from 91.4 to 97.1% for prostate cancer. The average age at death also increased but differed widely among the three cancer types. In 2017, it was 68.8 years, for breast cancer, 76.8 years, for lung cancer, and 80.7 years for prostate cancer. This increase also elevated the proportion of the total MtC for individuals aged ≥ 65 years. Among the three cancer types, the proportion of the MtC in individuals aged ≥ 65 was the highest for prostate cancer (82.0% in 2017), reflecting the higher proportion of individuals deceased over the age of 65 years. However, the MtC per death was the highest for breast cancer, being 5.7-fold greater than that for prostate cancer.

### Future projections

Table 3 shows the projected COIs for the three cancer types. Our model predicted that the COIs for breast and prostate cancers will continue to increase until 2029 (to 965.9 and 434.9 billion yen, respectively). Our model also showed a decrease in the COI for lung cancer after 2017, reaching 1,476.7 billion yen in 2029. The composition of costs in 2029 was predicted to remain similar to that in 2017. Thus, in 2029, the MtC for breast and lung cancers would account for approximately 60–70% of the total COI, whereas for prostate cancer, the DCs would reach approximately 70% of the total COI. Regarding contribution to the overall COI from 2017 to 2029, we found that the MtC accounted for the largest variation in the COIs for breast and lung cancers (+ 69.8 and − 61.9%, respectively). The DC was the largest contributor to the COI for prostate cancer (+ 111.3%).

**Table 3:** Future prediction of cost of illness (COI) for the three cancer types

		2017^a^	2020	2023	2026	2029
Estimated population (thousand persons)		126,706	124,100	122,122	119,891	117,465
% of individuals aged ≥ 65 years		27.7%	29.1%	29.8%	30.5%	31.2%
Breast cancer	Number of deaths (person)	14,423	15,504	16,435	17,322	18,288
	% of individuals aged ≥ 65 years	63.6%	63.9%	65.2%	66.3%	67.6%
	Average age at death (year)	68.8	69.3	70.2	70.9	71.7
	Direct cost (billion yen)	254.1	243.8	257.0	268.8	279.8
	Morbidity cost (billion yen)	56.9	51.5	51.1	50.6	49.9
	Mortality cost (billion yen)	592.7	618.3	625.7	632.5	636.2
	% of individuals aged ≥ 65 years	31.0%	30.6%	31.2%	31.9%	33.0%
	Mortality cost per person (million yen)	41.1	39.9	38.1	36.5	34.8
	COI (billion yen)	903.7	913.5	933.8	951.8	965.9
Lung cancer	Number of deaths (person)	73,883	80,969	83,547	84,630	87,727
	% of individuals aged ≥ 65 years	90.2%	90.2%	90.8%	91.1%	91.4%
	Average age at death (year)	76.8	77.5	78.2	78.6	79.3
	Direct cost (billion yen)	399.9	355.8	357.6	369.5	385.6
	Morbidity cost (billion yen)	52.3	47.9	43.9	41.2	39.5
	Mortality cost (billion yen)	1095.4	1135.6	1103.1	1070.1	1051.5
	% of individuals aged ≥ 65 years	63.7%	62.3%	62.9%	63.3%	64.0%
	Mortality cost per person (million yen)	14.8	14.0	13.2	12.6	12.0
	COI (billion yen)	1547.6	1539.2	1504.6	1480.9	1476.7
Prostate cancer	Number of deaths (person)	12,869	13,599	14,340	14,766	15,427
	% of individuals aged ≥ 65 years	97.1%	97.5%	97.7%	97.9%	98.0%
	Average age at death (year)	80.7	81.1	81.6	82.0	82.5
	Direct cost (billion yen)	279.6	283.8	297.5	314.1	328.6
	Morbidity cost (billion yen)	18.7	18.1	17.6	17.2	17.2
	Mortality cost (billion yen)	92.5	92.1	91.4	89.9	89.1
	% of individuals aged ≥ 65 years	82.0%	83.1%	83.8%	84.2%	84.8%
	Mortality cost per person (million yen)	7.2	6.8	6.4	6.1	5.8
	COI (billion yen)	390.8	394.0	406.6	421.2	434.9

Source of estimated population: National Institute of Population and Social Security Research “Population Statistics of Japan.”

^a^ Estimated using actual data

Our projected number of deaths increased for all three cancer types. Consequently, the estimated COI for 2029 increased to 18,288 (APC 2.0%), for breast cancer, 87,727 (APC, 1.4%) for lung cancer, and 15,427 (APC, 1.5%) for prostate cancer. The proportion of deceased individuals older than 65 years was predicted to be 67.6% for breast cancer, 91.4% for lung cancer, and 98.0% for prostate cancer. The average age at death was predicted to increase consistently to 71.7 years, for breast cancer, 79.3 years, for lung cancer, and 82.5 years, for prostate cancer. The contribution of patients aged ≥ 65 years toward the MtC also increased; however, it varied widely among the three cancer types: 33.0% for breast cancer, 64.0% for lung cancer; and 84.8% for prostate cancer. Overall, the MtC per death accounted for 34.8, 12.0, and 5.8 million yen, for breast, lung, and prostate cancers, respectively.

**Fig. 1:**
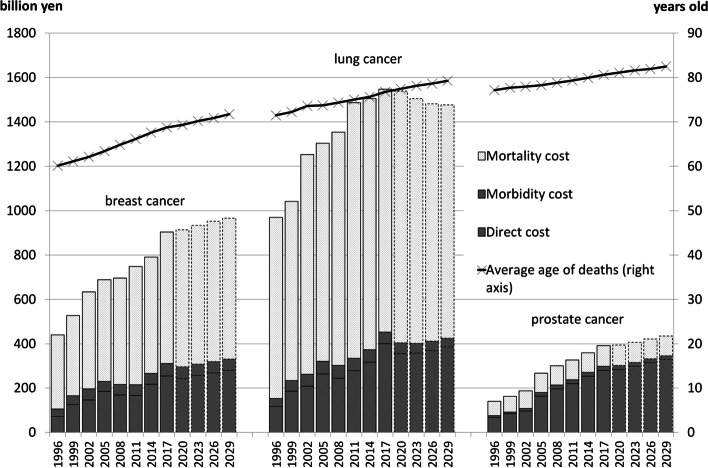
Cost of illness (COI) projection with cost elements and projection of the average age at death

Figure 1 shows the COI estimates for the 1996–2017 period, future projection to 2029, and the average ages at death. The COI for lung cancer was the highest and was estimated to peak in 2017. The COIs for breast and prostate cancers increased continuously throughout our projection. The average ages at death increased constantly for the three cancer types and did not change in order (breast cancer < lung cancer < prostate cancer).

### Human capital

To estimate the MtC, we added the present value of the annual income, discounted at a rate of 2%, to the annual monetary valuation of future unpaid work potentially earned until reaching the average life expectancy. According to the 2017 baseline data, the highest human capital was in 20–24 year-old men (137.1 million yen) and 25–29 year-old women (147.2 million yen). The human capital for the 65–69 year age group was 15.2% of the maximum value for men and 25.6% of the maximum for women.

### Sensitivity analysis by discount rate

The results of the sensitivity analysis for the past estimation and future projection are shown in Table 4. The change in the discount rate from 0 to 5% did not influence the trends observed in the COI.

**Table 4:** Sensitivity analysis by discount rate

	Discount rate	1996	1999	2002	2005	2008	2011	2014	2017	2020	2023	2026	2029
Breast cancer	0%	525.9	617.7	747.3	805.3	814.6	882.3	917.9	1054.2	1059.0	1074.8	1087.8	1094.4
	1%	478.8	568.0	684.7	740.9	749.2	808.7	847.7	972.5	976.4	993.3	1007.4	1015.8
	2%	440.3	527.2	633.7	688.3	695.7	748.5	790.2	905.8	908.7	926.2	941.2	950.9
	3%	408.4	493.3	591.6	644.8	651.3	698.7	742.4	850.6	852.6	870.5	885.9	896.7
	4%	381.8	465.0	556.5	608.5	614.1	656.9	702.3	804.4	805.5	823.6	839.4	850.8
	5%	359.2	440.9	526.9	577.8	582.6	621.6	668.3	765.4	765.6	783.9	799.9	811.8
Lung cancer	0%	1103.7	1169.9	1408.7	1459.1	1518.1	1667.2	1678.3	1728.1	1785.3	1758.1	1743.9	1750.5
	1%	1031.5	1100.9	1325.0	1375.5	1429.5	1570.3	1585.2	1637.9	1687.6	1663.8	1652.3	1660.9
	2%	969.6	1041.5	1252.7	1303.4	1352.9	1486.6	1504.5	1559.8	1602.8	1581.8	1572.7	1582.9
	3%	916.0	989.9	1189.9	1240.8	1286.2	1413.6	1434.0	1491.4	1528.8	1510.0	1502.8	1514.3
	4%	869.3	944.8	1134.8	1185.9	1227.7	1349.6	1372.0	1431.3	1463.6	1446.8	1441.1	1453.7
	5%	828.2	905.1	1086.2	1137.5	1176.1	1292.9	1317.1	1378.0	1405.9	1390.6	1386.3	1399.8
Prostate cancer	0%	123.2	141.7	182.8	258.2	298.2	340.2	369.2	399.4	410.0	424.0	440.0	455.1
	1%	120.7	139.1	178.8	254.2	293.6	334.8	364.1	394.2	404.6	418.7	434.7	449.9
	2%	118.5	136.9	175.2	250.6	289.6	330.0	359.5	389.5	399.7	413.9	430.0	445.3
	3%	116.5	134.8	172.0	247.4	285.9	325.7	355.4	385.2	395.3	409.5	425.7	441.0
	4%	114.7	133.0	169.2	244.5	282.6	321.7	351.7	381.4	391.3	405.6	421.8	437.1
	5%	113.1	131.3	166.5	241.8	279.6	318.1	348.3	377.9	387.7	401.9	418.2	433.6

## Discussion

Our results predicted a continuous increase in the COIs for breast and prostate cancers until 2029. The COI for lung cancer was predicted to peak in 2017. The MtC was the largest contributor to the COIs for breast and lung cancers, whereas the DC was the main contributor to the COI for prostate cancer. Among the three cancer types, the cost of mortality per person was the lowest for prostate cancer, as has been reported previously [44, 47].

For all three cancer types, we observed a clear trend toward an increasing number of deaths and an increase in the average age at death, which derive from the current aging trend in Japan. These two trends have opposite effects on the COI. An increase in the number of deaths directly increased the MtC, whereas an increase in the average age at death decreased the MtC by reducing human capital.

According to our projections, the average age at death will increase for the three cancer types. However, the cost structure profile will differ considerably among them. In 2017, the average age at death was 68.8 years for breast cancer, 76.8 years for lung cancer, and 80.7 years for prostate cancer. The human capital values for these groups were 25.6, 8.7, and 3.4% of their peak (baseline data), respectively, which are reflected in their respective MtC per capita. The average age at death for breast cancer was projected to increase by 2029 but to remain younger than that for the other two cancer types. Increased mortality outweighs the increase in the average age at death; thus, the MtC increases. Our annual labor estimates were based on 2017 data. However, as women’s participation in society (and consequently their income) increases, the MtC for breast cancer may increase above our projections. For lung cancer, the effect of an increase in the average age at death significantly reduces the human capital value per capita, which outweighs the effect of an increase in the number of deaths, resulting in a decrease in the MtC in 2017. Regarding prostate cancer, the baseline average age at death is in the late 70 s; therefore, the proportional contribution of the MtC to the total COI was low. The DC has been (and will be) increasing in the other two cancer types as medical care becomes more sophisticated and drugs more expensive, however, for prostate cancer, the percentage of the DCs was higher than in the other cancer types, which contributed significantly to the increase in the COI. Generally, with the increase in the DC, payments from Japanese public insurance institutions will increase. Public medical insurance and publicly funded medical providers bore 87.7% of the total medical expenditure in 2019.

We have shown some clear features of each of the three cancer types. As expected, the *young cancer* group (breast cancer) had the lowest average age at death. For this cancer, the impact of increased mortality outweighed the impact of reduced human capital, resulting in a higher COI. Cervical cancer also belongs to this group. For the *aging cancer* group (lung cancer), the COI remained greatly influenced by the MtC. However, at some point, the effect of the reduction in human capital will outweigh the effect of an increase in the number of deaths, when both the MtC and COI start to decrease. Stomach cancer also belongs to this group. The average age at death in the *aged cancer* group (prostate cancer) is already high and human capital is low. Consequently, the main factor affecting the COI is the DC.

Our results provide an evidence base for cancer control policies. For example, to reduce the COI for *young cancers*, research should seek to lower the mortality rate to reduce the MtC. For *aged cancers*, however, COI increases are directly related to the DCs; a reduced mortality rate could increase burden on public medical insurance, which could be contentious. The components of the COI are the DC, MbC, and MtC; however, each increase has different implications. The MtC and MbC are directly related to the health of the population, and these costs increase as the number of deaths or patients increases. Conversely, if the effectiveness of disease prevention measures and treatment increases, the ages at incidence and death are expected to increase further and morbidity and mortality costs are expected to decrease through a decline in the value of human capital. If DCs increase to produce a decrease in the MtC and MbC, the increase would be justified. Furthermore, although not reflected in the DCs, many resources used for disease control, such as government subsidies to promote medical research, should prioritize diseases expected to reduce the MtC and MbC. Therefore, on the micro level, it is important to take a multidisciplinary approach for cancer control and detailed response to the current situation of patients. On the macro level, it is important to enhance preventive measures for cancers, along with other aging diseases. Many previous studies have focused on this purpose [48–52]. Fortunately, previous studies have established the effectiveness of early vaccination and screening for cancers that occur earlier in life, such as breast and cervical cancers [53, 54].Allocating more resources to the fight against young cancers would make sense. Although COI research does not assess medical technologies, it can provide important information when considering the possible impact of introducing them.

This study has several limitations. First, our regression curves should be interpreted with caution. Our projections were based on data collected over a relatively short period, during which the healthcare system in Japan experienced major restructuring (e.g., introduction of a case-mix reimbursement system and differentiation between acute and nonacute beds). We set the annual labor value to that of 2017; however, women’s increasing income may increase the MtC for breast cancer beyond our projections. However, projections based on past trends are considered appropriate for drawing conclusions about the near future. To this end, our study suggests that the importance of breast cancer will increase in Japan within the next few years.

## Conclusions

We predicted a steady increase in the COI for breast cancer (a young cancer) due to the increase in MtC. However, the COI for lung cancer (an aging cancer) was projected to decrease due to the decline in MtC. The COI for prostate cancer (an aged cancer) was projected to increase due to the increase in DC. The identification of these trends can be useful for prioritizing of future policy development.

## Data Availability

All data used in the analysis are public data and can be obtained from the following website: https://www.e-stat.go.jp/.

## Abbreviations

COI: Cost of illness
ICD10: International Statistical Classification of Diseases and Related Health Problems 10th Revision
DC: Direct costs
IC: Indirect costs
MbC: Morbidity costs
MtC: Mortality costs
TOVy: Total person-days dedicated to outpatient visits
LVd: 1-Day labor value per person
NDy: Number of deaths
LVl: Lifetime labor value per person
THD: Total person-days of hospitalization
Iy: Annual income per person
ULVy: Annual monetary valuation of unpaid work per person
HPy: Annual number of hospitalized patients
ALOS: Average length of hospital stay
APC: Annual percent change
